# Posttraumatic Growth and Its Measurement: A Closer Look at the PTGI’s Psychometric Properties and Structure

**DOI:** 10.3389/fpsyg.2022.801812

**Published:** 2022-08-24

**Authors:** Bibiána Jozefiaková, Natália Kaščáková, Matúš Adamkovič﻿﻿﻿, Jozef Hašto, Peter Tavel

**Affiliations:** ^1^Olomouc University Social Health Institute, Palacky University, Olomouc, Czechia; ^2^Psychiatric-Psychotherapeutic Outpatient Clinic, Pro Mente Sana, Bratislava, Slovakia; ^3^Institute of Psychology, Faculty of Arts, University of Presov, Prešov, Slovakia; ^4^Institute of Social Sciences, Centre of Social and Psychological Sciences, Slovak Academy of Sciences, Košice, Slovakia; ^5^Department of Social Work, St. Elizabeth College of Health and Social Work, Bratislava, Slovakia

**Keywords:** posttraumatic growth, posttraumatic growth inventory, validation, network analysis, confirmatory factor analysis

## Abstract

Despite negative connotations, surviving trauma can result in improvements in some domains of a person’s life. This phenomenon is known as posttraumatic growth (PTG), and it is typically measured using the Posttraumatic Growth Inventory (PTGI). Given the ambiguous results of the existing validation studies, the present study aimed to verify the psychometric properties of the Slovak version of the PTGI in a representative sample of Slovak citizens. Although the results suggest that a modified one-factor structure fit the data best, other issues, such as extremely high correlations between the latent factors related to the PTGI’s factor structure, were observed. It is likely that the application of the latent variable model does not represent the essence of PTG adequately and the network approach thus appears to be a far more suitable conceptualization of PTG. More detailed information on between-person differences and within-person changes in PTG could help to tailor more effective interventions or preventive programs.

## Introduction

Approximately 70% of people experience at least one potentially traumatic event in their lifetime (Benjet et al., 2016; Knipscheer et al., 2020). Based on the criteria stated in the DSM-5, trauma is defined as an event in which a person is exposed to actual or imminent death, severe injury or sexual violence (American Psychiatric Association, 2013). The word “trauma” is usually perceived negatively, and the majority of research on this topic is focused on its negative consequences on mental health. How a person responds to surviving a traumatic event depends on multiple factors (biological, psychosocial, etc.). PTSD is just one of many possible types of reactions (Yehuda et al., 2015), and it develops in about 5% of people who survive a traumatic event (Atwoli et al., 2015). The prevalence rates stem from a combination of historical circumstances (e.g., war/conflicts, high criminality rates, natural disasters, etc.; see Asnakew et al., 2019) and the mental health care infrastructure within individual countries, which determines the practices related to PTSD diagnosis and treatment.

After trauma, a prototypical pathway of recovery can be observed. First, an elevation of psychological symptoms with poor functioning for at least several months occurs before they return to baseline, pre-trauma levels. Bonanno (2004) assumes that individuals with a typical recovery trajectory after trauma are most likely to experience and report some positive consequences of the trauma for their lives. Westphal and Bonanno (2007) argue that more resilient people tend not to struggle with some potentially traumatic events to the same extent as might other, more traumatized individuals. There are many controversies between the concepts of PTG and resilience. Hobfoll et al. (2015) implicitly equate posttraumatic growth with resilience or view it as a superior construct covering resilient outcomes. In reaction to this suggestion, Westphal and Bonanno (2007) argue that many if not most people are resilient in the face of trauma and that resilient outcomes typically provide little need or opportunity for PTG. This is supported by a growing number of prospective studies that have demonstrated that many (often the majority of) people exposed to potentially traumatic events exhibit a stable resilient outcome trajectory and are significantly less likely to search for meaning following some loss or potential trauma compared to others exposed to the same event. However, in the last 25 years, research has also started to focus on the positive consequences of surviving a traumatic event. Different terms have been used to describe positive psychological changes after surviving a potentially traumatic event, for instance, positive psychological changes (Yalom and Lieberman, 1991), stress-related growth (Park et al., 1996), flourishing (Ryff and Singer, 1998) or adversarial growth (Joseph, 2009).

The most cited and most elaborated is the theory of posttraumatic growth (PTG; Tedeschi and Calhoun, 1996). PTG is defined as a positive change in certain areas of life as an aftermath of trauma. A positive psychological change can happen in (at least) one of the following domains: (1) interpersonal relationships, (2) new possibilities, (3) personal strength, (4) spiritual change and (5) appreciation of life (Tedeschi and Calhoun, 1996). The phenomenon of PTG has been conceptualized as an outcome of the struggle with a traumatic event or as a coping strategy (Zoellner and Maercker, 2006). PTG has been mostly studied in samples of war veterans (Mark et al., 2018), survivors of a natural disaster (García et al., 2015), victims of sexual violence (Bakaityté et al., 2020), oncological patients or people diagnosed with other serious conditions (Hamama-Raz et al., 2019). Working with such specific groups could have narrowed the focus of research on traumatic events to the most extreme ones (e.g., war, natural disaster) despite the fact that people experience a wider range of traumatic events throughout their lives (Brooks et al., 2016). According to Kessler et al. (2017), the three most burdensome traumatic events are sexual violence (15.1%), rape (13.1%) and the unexpected death of a loved one (11.9%). Mills et al. (2011) found that the most frequently experienced events among men was having seen someone being badly injured or killed or having unexpectedly seen a dead body; among women it was having had someone close die unexpectedly.

Various research findings have emphasized that the variation of PTG is determined by potentially traumatic/traumatic life events experienced by the person (e.g., Lowe et al., 2020). Some authors have argued that events driven by natural processes (natural disasters or disorders) are related to major posttraumatic growth than those with human interactions, such as sexual violence (e.g., Ickovics et al., 2006; Meyerson et al., 2011). On the other hand, according to some authors (e.g., Ulloa et al., 2016), traumatic events that are related to some kind of sexual violence may lead to growth because of their major effect on the survivors’ self-perception and their potential awareness of social themes related to their experience. According to a meta-analysis by Wu et al. (2019), about 53% of people exposed to some form of a traumatic event (chronically ill people, war veterans, firefighters, rescuers, etc.) consequently experience at least medium posttraumatic growth, with women reporting higher scores of PTG than men (e.g., Vishnevsky et al., 2010; Hamama-Raz et al., 2020). However, gender differences in PTG scores seem to depend on the measure used to examine PTG (Barskova and Oesterreich, 2009).

Although researchers from different countries have confirmed that PTG is universal (e.g., Netherlands: Jaarsma et al., 2006; Germany: Mack et al., 2015; China: Xu et al., 2021), some evidence suggests the existence of cultural differences that may be observed in PTG (e.g., Exenberger et al., 2019). The major reason is the fact that different cultures may explain the word “trauma” in different ways and may react to it differently (e.g., Kashyap and Hussain, 2018). These differences may also be attributed also to the differences between individualistic and collectivist cultures (e.g., Kashyap and Hussain, 2018).

With more than 7,000 citations, the Posttraumatic Growth Inventory (PTGI) is the most frequently used method for measuring PTG. The items in the original version of the questionnaire were mainly based on the authors’ interviews with people who had severe physical difficulties or had experienced the death of a loved one (husband/wife), and the questionnaire was validated on a sample of students (Tedeschi and Calhoun, 1996). There are currently three other versions of the original questionnaire: its shortened form (PTGI-SF; Cann et al., 2010), a version for children (PTGI-C; Cryder et al., 2006) and a version with an expanded spiritual-existential change scale (Tedeschi et al., 2017). According to the authors of the original version, the questionnaire consists of five subscales that represent the five PTG domains described above. Besides calculating a score for each subscale, a summary score can be derived (Tedeschi and Calhoun, 1996). The questionnaire has been validated by several research teams from different countries (e.g., Joseph et al., 2004; Jaarsma et al., 2006; Linley et al., 2007; Osei-Bonsu et al., 2012; Mack et al., 2015; Arandia et al., 2018; Silverstein et al., 2018; Xu et al., 2021). The results of these validation studies pointed to inconsistencies in the factor structure of the PTGI questionnaire. While some studies have supported the original five-factor structure of the questionnaire (e.g., Ramos et al., 2016), other authors have observed the best fit for a three-factor structure (e.g., Rodríguez-Rey et al., 2016), a four-factor structure (e.g., Pajón et al., 2020), or structures with multiple latent factors in general (e.g., Osei-Bonsu et al., 2012). Besides the natural variations caused by language/culture adaptation and sampling, the results may differ due to the use of different statistical procedures to verify the factor structure of the PTGI. In practice, however, either the initial five-factor model or the general one-factor structure (i.e., a simple summary score) is widely used (Steffens and Andrykowski, 2015).

Based on the PTG theory described by Tedeschi and Calhoun (1996), spirituality is considered to be the main aspect of PTG. Spirituality has been found to mediate the path between trauma and PTG in parents who have lost their young children (Khursheed and Shahnawaz, 2020). In the study of patients with breast cancer, spirituality predicted higher PTG (Paredes and Pereira, 2017).

Because of (1) the unclear factor structure of the PTGI and (2) the fact that validity of the original version of the measure does not guarantee that its adaptation to other languages will be valid as well (see, e.g. Byrne, 2016), the main aim of this study was to verify the psychometric properties of the Slovak version of the Posttraumatic Growth Inventory (PTGI) on a representative sample of Slovaks. The additional goals of this study were to examine the invariance of the instrument across gender and to examine its relations with external variables (spirituality and resilience) and also with the type of traumatic or stressful event. As spirituality is one of the areas of potential growth, we expect PTG to be positively correlated with spirituality, and we expect moderate relationships between these two variables (e.g., Paredes and Pereira, 2017; Khursheed and Shahnawaz, 2020). Based on the framework of Westphal and Bonanno (2007) that more resilient people provide little opportunity for PTG, we hypothesized that resilience will be strongly and negatively correlated with PTG (Levine et al., 2009; Ying et al., 2016; Zhang et al., 2019).

## Materials and Methods

### Participants and Data Collection

Data was collected in April 2019. Based on quota characteristics (gender, age, education, size of the place of residence, and region of residence), a total of 1018 respondents were selected. Quota characteristics were calculated based on data from the Statistical Office of the Slovak Republic. More descriptive data about the sample are available in Tables 1, 2. Using the Life Stressor Checklist (LSC-R), we identified that 71% (*N* = 721) of participants in the sample had survived a traumatic or stressful life event. Those participants were then administered the Posttraumatic Growth Inventory (PTGI). The study was approved by the Ethics Committee of the Olomouc University Social Health Institute, Palacky University Olomouc (No. 2019/05).

**Table 1:** Demographic characteristics of the sample.

	*N*	%
**Gender**		
Male	496	48.7
Female	522	51.3
**Age**		
18–24 years	110	10.8
25–34 years	187	18.4
35–44 years	199	19.5
45–54 years	166	16.3
55–64 years	168	16.5
65 or more	188	18.5
**Living with**		
A partner	671	65.9
Alone	162	15.9
Parents	185	18.2
**Level of education**		
Primary school	137	13.5
Secondary vocational school	272	26.7
High school	382	37.5
University degree	227	22.3

**Table 2:** Prevalence of different types of stressful and traumatic events in the whole sample (*N* = 1,018).

Stressful/traumatic event	*N* (%)
Natural disaster (earthquake, hurricane, explosion)	102 (10%)
Serious accident—witness (e.g., car wreck)	209 (20.5%)
Serious accident	53 (5.2%)
Incarceration of a family member	33 (3.2%)
Incarcerated	2 (0.2%)
Own adoption	5 (0.5%)
Separation/ divorce of parents	99 (9.7%)
Own separation/divorce	95 (9.3%)
Financial difficulties (e.g., not enough money for food or place to live)	186 (18.3%)
Serious physical / mental illness (e.g., cancer, heart attack)	62 (6.1%)
Emotional abuse (e.g., frequently shamed, embarrassed, ignored, etc.)	49 (4.8%)
Physical neglect (e.g., not fed, not properly clothed, etc.)	29 (2.8%)
Induced abortion	29 (5.5% of woman)
Miscarriage	57 (11% of woman)
Difficult birth	37 (7% of woman)
Separation from own child (e.g., loss of custody or visitation or kidnapping)	7 (0.7%)
Severe physical or mental handicap of a child (e.g., mentally retarded, birth effects etc.)	15 (1.5%)
Caring for a loved one with a disability	72 (7.1%)
Unexpected death of a loved one (e.g., sudden heart attack, murder, suicide)	273 (26.8%)
Death of a loved one	383 (27.6%)
Witness of family violence—before the age of 16 (e.g., hitting, kicking, punching etc.)	69 (6.8%)
Robbery—witness	26 (2.6%)
Have been robbed	26 (2.6%)
Physical abuse before the age of 16 by someone they knew	89 (8.7%)
Physical abuse after the age of 16	25 (2.5%)
Sexual harassment	37 (3.6%)
Sexual touching before the age of 16	9 (0.9%)
Sexual touching after the age of 16	6 (0.6%)
Forced sex before the age of 16	8 (0.8%)

To get a better grip on the prevalence rates of each type of trauma in the general Slovak adult population, the sample in this table is not limited to the participants who experienced a traumatic event.

### Measures

#### Posttraumatic Growth Inventory (PTGI)

The PTGI measures the level of posttraumatic growth in persons who have survived a traumatic event (Tedeschi and Calhoun, 1996). It consists of 21 items, each of which falls under one of the five factors: (1) relating to others, (2) new opportunities, (3) personal strength, (4) spiritual change and (5) understanding of life. Participants are asked to indicate the degree to which they have or have not experienced a particular change using a scale ranging from 0 to 5. A higher score indicates a higher level of posttraumatic growth. Examples of items: (1) I’m more aware that I can handle difficulties, (2) I’m putting more effort into my relationships or (3) I’ve found out how great people are. The PTGI does not measure specific changes in behavior, but subjectively evaluated changes in the concept of the world, relationships with other people, and the self. The Slovak version of the PTGI was created by two independent experts in the field of psychotraumatology and one psychologist, then back-translated into English by a licensed translator. All versions were compared and discussed and a consensus on the final version was reached. The reliability of the whole scale (one-factor) was ω_total_ = 0.98, while the reliabilities of the subscales ranged from ω_total_ = 0.86 to 0.96.

#### Brief Resilience Scale (BRS)

Resilience was measured using the Brief Resilience Scale (BRS; Smith et al., 2008, the Czech and Slovak validation was done by Furstova et al., 2021). The BRS consists of six items and measures resilience as the ability to recover from a stressful event. Examples of items: (1) It is difficult for me to go through a stressful situation or (2) I tend to recover quickly from difficult situations. The reliability of the scale was ω_total_ = 0.87.

#### Spiritual Well-Being Scale

The Spiritual Well-Being Scale (SWBS; Paloutzian and Ellison, 1982) is a self-report questionnaire that measures spiritual and life well-being. The Slovak version of SWBS was validated by Tavel et al. (2022). The SWBS consists of 20 items, from which either a summary score can be calculated or two subscales (religious well-being and existential well-being) can be derived. In this sample, the scale showed high reliability with ω_total_ = 0.87. Examples of items: (1) I don’t know who I am, where I came from, or where I’m going or (2) I believe that God is concerned about my problems.

#### Functional Assessment of Chronic Illness Therapy—Non-illness Version (FACIT-Sp-12)

FACIT is a self-report questionnaire designed to measure spiritual well-being and quality of life. It was initially designed for people diagnosed with serious diseases (Peterman et al., 2002). In our study, its 12-item non-illness version designed for the general (healthy) population (where the word “disease” was changed to the word “difficult time”) was used. This version was first validated as a 23-item version, the FACIT-Sp-Ex (Brintz et al., 2017). Four items of the questionnaire focus on the meaning and purpose of life, four items on inner peace, and four items on faith. The reliability of the whole scale is high with ω_total_ = 0.88. Examples of items: (1) I feel peaceful, (2) I have a reason for living or (3) My life lacks meaning and purpose.

#### Life Stressor Checklist

The prevalence of exposure to life stressors was assessed using the Life Stressor Checklist Revised (Wolfe et al., 1996). The LSC-R is a 30-item questionnaire; 19 items assess events that have a potential for psychological trauma, and nine items focus on other stressful life events. Additional questions provide insights into the age of the person at the time of surviving the event, if survivors experienced intensive fear, helplessness or fear for their life during the event, and how much this situation affects them in later life. The different scores of the LSC-R also comprise the subjective burden of the individual stressor and its impact on the actual life (Kaščáková et al., 2018).

### Statistical Analysis

In the initial screening, descriptive statistics were calculated, and the data were screened for missing and improbable values. Given the online administration, the items did not contain any missing values or values that were unlikely to occur (e.g., typos)—all the observed values were within the range of the response scales. As such, no observations were considered as outliers and no transformation of data was applied (note: as a part of the sensitivity analysis, we reproduced the analytic flow after the exclusion of the participants with a Mahalanobis distance > 3 SD and obtained essentially the same results as reported below). The initial screening also included an inspection of the correlation matrix of the PTGI items.

Afterwards, the dataset was randomly split into two parts—an exploratory (N_E_ = 360) and a confirmatory (N_C_ = 361) part. *A priori* analysis of the statistical power based on the RMSEA coefficient (α = 0.05; H_A_ RMSEA = 0.08; H_0_ RMSEA = 0.04) indicated that for a combination of a sample of *N* = 360 and a model with df = 179 (the five-factor model), the statistical power to detect the model’s misspecification converges to 100%. Although both PTGI models were constructed in accordance with the conventions of PTG research (Tedeschi and Calhoun, 1996; Silverstein et al., 2018), the form of cross-validation used here was preferred, as the occurrence of some misspecifications was expected. The exploratory dataset served to address these misspecifications (all the potential modifications had to be, first and foremost, theoretically justifiable). The confirmatory dataset was hence used to cross-validate the results and to select the most optimal PTGI structure. Consequent invariance testing (with gender as a potential source of invariance) and examination of convergent validity was performed only for the best-fitting model. When examining convergent validity, the external variables (BRS, SWBS, and FACIT) were modeled together in one general model (which, obviously, also included PTGI), and correlations between the latent variables were calculated. From the technical perspective, the models were initially estimated using the WLSMV method, with the items being treated as ordinal. The models were also fitted using the maximum likelihood (ML) estimator due to the technical problems with fitting the five-factor model using the WLSMV (i.e., a Heywood case with a correlation coefficient between the factors exceeding 1; see Results), as well as for the purpose of comparison of competing models using the chi-square difference test. Had the value of chi-square been significant, the models would be considered disconfirmed (note, the chi-square test is the only statistical test of model-data fit in structural equation modeling (SEM); that is, it tests the exact-fit hypothesis that there is no difference between the covariance matrix implied by the model and the matrix of the observed covariances; see Ropovik, 2015; Kline, 2016) been significant. The potential sources of the models’ misfit were inspected (factor loadings, covariances between latent factors, residual matrix and modification indices were checked). Apart from calculating chi-square values, the fit of the models was diagnosed using the (scaled) conventional approximate fit indices (AFI), namely, CFI, TLI, RMSEA and SRMR. The satisfactory values, indicating a good local fit of the model, were set to 0.95 for CFI and TLI, 0.06 for RMSEA and 0.08 for SRMR (see Hu and Bentler, 1999). Given the nested structure of the tested models (the expected modified one-factor model), a formal chi-square difference test was calculated to determine which of the models fit the data best. To examine the reliability of the respective factors, McDonald’s omega (utilizing polychoric correlations) was computed.

#### *Post-hoc* Analysis

With regard to the problematic (e.g., correlation coefficients between the latent factors equal to or exceeding 1) and hardly interpretable results of the performed confirmatory factor analyses (see Results below), a network analysis was calculated (as an exploratory part of this study) for the whole dataset. The traditional, more or less implicit assumption of a latent variable that causally determines the observed (measured) behaviors is, in fact, only barely justifiable for the conceptualization of psychological constructs (e.g., latent variable models assume causality flowing from the latent variable to the observed indicators, local independence of the indicators after controlling for the latent variable, or that an indicator-level intervention cannot have an effect on the latent variable; see, e.g., Borsboom, 2008; Borsboom and Cramer, 2013; Schmittmann et al., 2013). To the contrary, PTG (or, eventually, any other psychological construct; see e.g., Borsboom and Cramer, 2013) is composed of a set of indicators that are mutually connected and have an inner structure. Instead of assuming the existence of a latent variable, in this approach, the system of causally related variables that “hang” together ultimately represents the construct, overcoming the above-mentioned caveats of traditional latent models. The network approach reveals the structure of a psychological construct by estimating which indicators play a more central/peripheral role and how the indicators are interconnected. Conceptually, this approach can be seen as more realistic compared to other attempts to improve the fit of models (e.g., testing second-order factor models). With respect to the goals of this paper, computing a network of the PTGI items had not been initially intended. Thus, the below-presented network has more of a demonstrative (rather than technically rigorous) purpose. The network was estimated using the EBICglasso estimator (the tuning parameters were set to prefer a sparser network). Centrality/connectivity parameters, as well as indices of network stability and replicability, were calculated. The analyses were performed in R (R Core Team, 2020), with *psych* (Revelle, 2020), *lavaan* (Rosseel, 2012), *qgraph* (Epskamp et al., 2012) and *bootnet* (Epskamp et al., 2018) serving as the main packages.

## Results

A correlation matrix, as well as means and standard deviations of the PTGI items, is available in Table 3.

**Table 3:** Correlation matrix and means and standard deviations of the PTGI items.

	1	2	3	4	5	6	7	8	9	10	11	12	13	14	15	16	17	18	19	20	21
1	-	0.81	0.70	0.65	0.57	0.61	0.70	0.62	0.60	0.66	0.71	0.68	0.69	0.64	0.63	0.62	0.65	0.47	0.68	0.60	0.64
2		-	0.73	0.68	0.61	0.68	0.70	0.67	0.63	0.68	0.74	0.73	0.74	0.66	0.69	0.66	0.69	0.48	0.71	0.63	0.68
3			-	0.76	0.55	0.64	0.76	0.65	0.62	0.65	0.69	0.67	0.65	0.70	0.64	0.64	0.66	0.44	0.65	0.63	0.64
4				-	0.57	0.68	0.72	0.69	0.68	0.71	0.72	0.71	0.67	0.70	0.64	0.67	0.69	0.45	0.71	0.68	0.66
5					-	0.65	0.62	0.63	0.59	0.59	0.61	0.61	0.59	0.56	0.62	0.52	0.56	0.73	0.60	0.57	0.64
6						-	0.67	0.76	0.68	0.71	0.72	0.73	0.68	0.66	0.70	0.66	0.67	0.51	0.67	0.71	0.74
7							-	0.74	0.68	0.70	0.73	0.70	0.68	0.73	0.64	0.68	0.70	0.48	0.70	0.66	0.68
8								-	0.78	0.71	0.72	0.73	0.69	0.69	0.76	0.73	0.70	0.53	0.68	0.72	0.75
9									-	0.72	0.70	0.70	0.67	0.67	0.70	0.73	0.70	0.50	0.67	0.67	0.71
10										-	0.86	0.82	0.78	0.72	0.73	0.70	0.74	0.46	0.78	0.69	0.75
11											-	0.86	0.82	0.75	0.76	0.74	0.76	0.49	0.79	0.73	0.77
12												-	0.81	0.73	0.74	0.72	0.76	0.49	0.77	0.72	0.77
13													-	0.72	0.76	0.73	0.75	0.51	0.74	0.70	0.75
14														-	0.74	0.68	0.74	0.48	0.72	0.70	0.72
15															-	0.73	0.75	0.55	0.72	0.73	0.81
16																-	0.80	0.45	0.68	0.70	0.74
17																	-	0.49	0.75	0.73	0.78
18																		-	0.57	0.54	0.57
19																			-	0.76	0.77
20																				-	0.84
21																					-
M	2.22	2.54	2.14	2.19	1.91	2.22	2.11	2.09	2.02	2.31	2.41	2.35	2.61	2.02	2.30	2.22	2.15	1.66	2.32	2.21	2.20
SD	1.58	1.58	1.52	1.48	1.54	1.49	1.57	1.45	1.46	1.51	1.54	1.48	1.58	1.55	1.45	1.45	1.48	1.59	1.57	1.50	1.44

### Confirmatory Analyses Results

#### Exploratory Dataset

The original one-factor model (χ^2^(47) = 686.96; *p* < 0.001), as well as the original five-factor model (χ^2^(48) = 481.55; *p* < 0.001) showed significant deviations from data. The mean value of the factor loadings was very high—λ = 0.86 (ranging from 0.68–0.94) for the one-factor model and λ = 0.89 (ranging from 0.79–0.96) for the five-factor model, respectively. In the one-factor model, there were observed high residual covariances (cov > 0.10) between the items no. 1 and 2, 3 and 4, and 5 and 18. Modification indices (MI > 10) suggested that a covariance term between 14 pairs of the items be included, with the absolute values of the standardized expected parameter change ranging from 0.25 to 0.78. After adding a covariance term between the theoretically justifiable pairs of the items (items no. 5 and 18, 20 and 21, 1 and 2, 10 and 11, 11 and 12, 3 and 4, and 16 and 17), the value of the chi-square statistics dropped substantially. The model was, however, still deemed disconfirmed. Despite showing a much better fit compared to the one-factor model, the five-factor model flagged problems with the convergence – the correlations between the five latent factors were extremely high (the mean correlation was 0.89), and the estimated correlation between factor 2 and factor 3 exceeded the value of 1. The estimation of the model using ML had still produced similar problems with its convergence. Although there were no visible problems with residual covariation in this case, the values of modification indices were erroneous. Due to high collinearity between the factors, the factors correlating above 0.8 were merged, creating a model with two latent factors (items no. 5 and 18 loaded on one factor and all the other items loaded on the second factor). Even though the two-factor model converged, sources of its misspecifications were similar to those observed in the one-factor model. The comparison of the nested model using the likelihood ratio test showed that, out of the candidate models, the five-factor model fit the data best. When compared with the non-nested modified one-factor model (probabilistic model selection), the five-factor model showed worse values of information criteria AIC and BIC. The parameters of models’ fit are summarized in Table 4. The reliabilities of the respective subscales (both in the one-factor the two-factor and the five-factor model) were extremely high ω_Total_ = 0.90–0.98, except for the reliability of the fourth factor in the five-factor model, which was still very high (ω_Total_ 0.86).

**Table 4:** Model fit parameters (scaled) for both the exploratory and the confirmatory dataset.

Model	*χ* ^2^	df	Value of *p*	CFI	TLI	RMSEA [95% CI]	SRMR
Exploratory dataset							
One-factor	686.96	47	<0.001	0.87	0.99	0.20 [0.18, 0.21]	0.04
Five-factor	481.55	48	<0.001	0.91	0.99	0.16 [0.15, 0.17]	0.03
Modified one-factor	381.62	50	<0.001	0.93	0.99	0.14 [0.12, 0.15]	0.03
Two-factor	603.53	47	<0.001	0.89	0.99	0.18 [0.17, 0.19]	0.04
Confirmatory dataset							
One-factor	713.25	37	<0.001	0.87	0.98	0.23 [0.21, 0.24]	0.05
Five-factor	438.70	37	<0.001	0.92	0.99	0.17 [0.16, 0.19]	0.04
Mod. one-factor	379.60	38	<0.001	0.94	0.99	0.16 [0.15, 0.17]	0.04
Two-factor	536.81	37	<0.001	0.91	0.99	0.19 [0.18, 0.21]	0.04

#### Confirmatory Dataset

All the models tested in the exploratory dataset were fitted again in the confirmatory dataset. Similar to the previous results, the five-factor model had troubles with convergence regardless of the estimation method, as there was extremely high collinearity between the latent factors. Given that the modified one-factor model had fit the data best, it was used for further analysis of convergent validity and measurement invariance across gender. The fit parameters for all the models are presented in Table 3.

Assuming potential gender differences in the PTGI structure, measurement invariance was calculated on the modified one-factor model. The results indicate that the PTGI structure is invariant across gender in term of metric (Δχ^2^ = 20.92, Δdf = 20, *p* = 0.402) and scalar (Δχ^2^ = 28.34, Δdf = 20, *p* = 0.102) invariance. Significant non-invariance was observed in terms of the latent means of PTG (Δχ^2^ = 10.12, Δdf = 1, *p* = 0.001), with women scoring 0.35 SD (converted to the PTGI scale = 9.1 points) higher than men.

The modified one-factor model was then correlated with the BRS (*r* = −0.05), SWBS (*r* = 0.29) and FACIT (*r* = 0.32) within one general model. The reported coefficients thus represent correlations between the latent variables. It is worth noting that item no. 18, which describes religious faith, showed very high cross-loadings on both the SWBS and FACIT. For descriptive purposes, the one-factor model was correlated with the Life Stressor Checklist (LSC-R), which serves as a screening for stressful (traumatic) experiences. The results of the LSC-R were then divided into five scores: (1) interpersonal violence, (2) indirect trauma, (3) traumatic events experienced before 16 years old, (4) a summary score of traumatic exposure and (5) other forms of trauma (see Schumacher et al., 2010; Kaščáková et al., 2018). The observed correlations between the PTGI score and the above-described scores were *r* = 0.10, *r* = 0.22, *r* = 0.15, *r* = 0.22, and *r* = 0.23 for interpersonal violence, indirect trauma, traumatic events experienced before 16 years old, summary score of traumatic exposure and other forms of trauma, respectively. When assessing individual events, the strongest correlations were found between the PTGI score and being directly involved in or a witness to an accident (*r* = 0.33; *r* = 0.18), taking care of a long-term sick relative (*r* = 0.31), and an unexpected death or the death of loved ones (*r* = 0.26; *r* = 0.29).

### PTG as a Network

**Figure 1:**
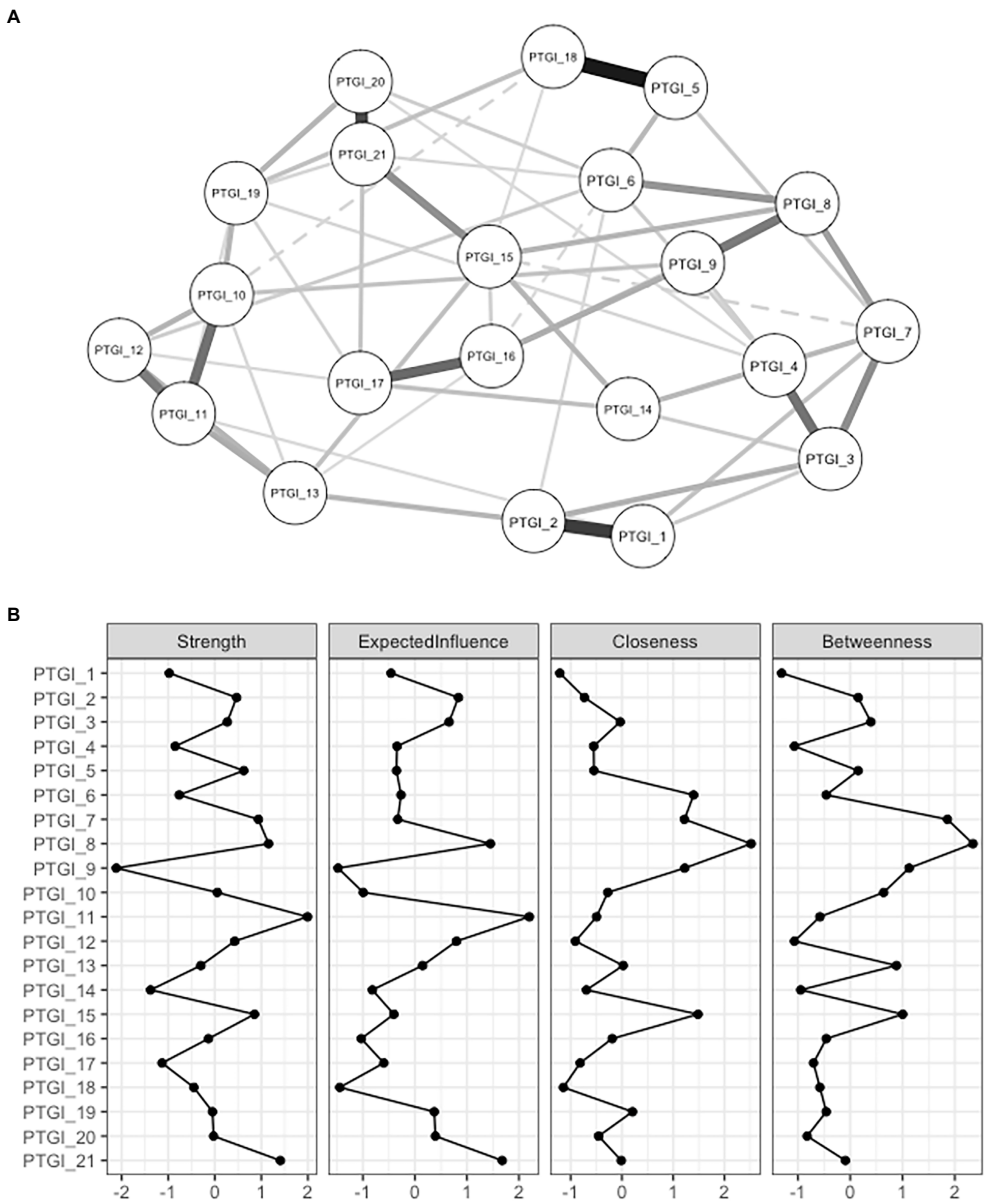
Visualization of a network of the PTGI’s items and their centrality indices.

The performed analyses point to five main conclusions: (1) there was a strong correlation (average *r* = 0.68) between the PTGI items; (2) all the PTGI items loaded very well on the latent factor/factors; (3) if PTGI was modeled with more than one latent factor, there were extremely high correlations between the factors; (4) the modified one-factor model fit the data best; and (5) none of the PTGI items was problematic *per se*. The combination of these facts suggests that it is next to impossible to find an optimal factor structure for the PTGI (note: this assumes that all the PTGI items are important for the operationalization of PTG—in other words, the items in PTGI capture all the theoretically relevant aspects of PTG). It is therefore possible that the inability to find an optimal factor structure stems from the way PTG is statistically modeled in validation studies, including this one. Applying reflective latent models to psychological constructs rests on several assumptions which are usually not tenable. For example, in reality, it is unlikely that an underlying latent variable (or, say, five latent variables) exists representing PTG that causes its observable indicators. Rather on the contrary, mutual relationships between the specific behavioral aspects (indicators) and their dynamics cause a system which is conventionally labeled as PTG. This radical shift in the perspective and understanding of a phenomenon (from latent models to a network approach) subsequently changes the nature of the research questions being asked; for instance: (1) how are the indicators of PTG related? (2) Which PTG indicators play a core, or on the other hand, a peripheral role in the system? To provide insight into these emerging questions, a network consisting of PTG indicators was estimated. A visualization of this (conservative—small edges were shrunk to zero) network is presented in Figure 1A.

As can be seen from Figure 1B, the highest degree of centrality (strength of a direct association between an indicator and other indicators; strength and expected influence) was found for items no. 11, 21 and 8. The highest connectivity, in terms of how strongly a node is indirectly connected with other nodes (closeness), was found for items no. 8, 6, 7, 9 and 15, while items no. 7 and 8 were the most important for connecting other nodes (betweenness).

The estimated network is relatively robust. Bootstrapped estimates suggest that the above-described parameters are rather stable, although the stability is not ideal (for all the parameters, the point estimate of average correlation with the original sample is above *r* = 0.50 even when 50% of cases are dropped).^1^ The presented network is also fairly replicable. Estimates obtained from replicating and simulating the network highly correlate (*r* ≈ 0.70 and higher) with the original values (except for Jaccard index), even when 250 cases are sampled. Altogether, the network performs well enough to make some initial inferences; nonetheless, future replication studies are very much needed.

## Discussion

The aim of this study was to verify the factor structure of the Slovak version of the Posttraumatic Growth Inventory (PTGI) on a representative sample of the Slovak population. The analyses revealed strong correlations between the PTGI items and also extremely strong correlations between the latent factors (had the PTG model included more than one latent factor). The results suggested that a modified one-factor model overperformed the competing models. While the structure of the one-factor model was invariant across gender, a difference in the latent means was observed (women scored higher compared to men). The questionnaire is thus applicable to both men and women (Mordeno et al., 2015). The convergent validity of the modified one-factor model of the PTGI was examined by correlating the factor with the external criteria spirituality (FACIT-Sp-12 non-illness, SWBS) and resilience (BRS). A weak to moderate positive relationship with spirituality and a weak negative relationship with resilience were observed. Similar findings were found in other studies (Shaw et al., 2005; Danhauer et al., 2013; Paredes and Pereira, 2017).

Although the modified one-factor model fit the data best, altogether, the observed results (e.g., even the best-fitting model significantly deviated from the data; very high correlations between the items; extremely high correlations between the PTGI subscales, if the model consists of more than one latent factor) suggest that the main issue may lie elsewhere. Given the observed results, but also the conceptual basis for measuring psychological constructs, applying the latent variable model (a latent factor is a single cause of the observed/reported indicators) to PTG might not be appropriate. Instead, a network approach, in which PTG is regarded as a set of mutually interacting indicators that form a structure consensually labeled as PTG, is a more appropriate representation (for similar argumentation in PTSD research, see Armour et al., 2017).

The existing evidence (e.g., Silverstein et al., 2018), as well as the present results (high correlations between latent factors), suggest that although posttraumatic growth as a construct can be observed in different domains of life (Tedeschi and Calhoun, 1996), it is probably the same variable. Dividing the PTGI into factors could potentially help to better capture the nature of this phenomenon from the theoretical perspective. The empirical evidence, however, suggests that this distinction is rather didactical. If a researcher aims to study PTG, a reduction in the number of administered items (e.g., administering a short form of the PTGI, see Cann et al., 2010; Lamela et al., 2014) could save resources as well as the participants’ time and effort, subsequently leading to a higher quality of the obtained data. Therefore, if an item is not essential with respect to the constitutive definition of PTG, removing it from the measure might be worth consideration. In other words, it might be useful to take a step back and look at the constitutive definition of PTG and utilize the corresponding operationalizations in the questionnaire.

Shifting the perspective from a latent variable model to the more structured network approach would allow detecting the central/peripheral indicators. The indicators showing high centrality indices are theorized to be good intervention targets, as they are the most closely related to all the other indicators in the network and, as such, are more likely to influence the development of the other indicators within the network (e.g., Levinson et al., 2018). On the other hand, indicators that are low on centrality/connectivity indices are less likely to be influential for the network. Identification of the roles of the variables forming a construct helps to design interventions/facilitation strategies. For dynamic systems, tailoring interventions solely from cross-sectional data could be tricky (see Rodebaugh et al., 2018; Henry et al., 2020) and more longitudinal research and studies that use experience sampling will be needed. Based on the performed calculations, items no. 6, 7, 8, 9, 11, 15 and 21 appear to be the most central ones. These items correspond to the first (relating to others) and the second (new possibilities) factor from the original five-factor structure. Based on this, we can consider the quality of relationships and the social environment a person has, how they are able to communicate their difficulties and how their view of life and confidence in their own coping skills will changeto be the most important for posttraumatic growth. Bellet et al. (2018) have already examined a network structure of PTG. They, however, used the short form of the PTGI (PTGI-SF) and were primarily focusing on the co-occurrence of PTG and complicated grief. According to their findings, the core indicators (based on the expected influence measure) of PTG were the items no. 6 and 9 (corresponding to items no. 7 and 19 in the original, full version of the PTGI questionnaire). These items fall under the new possibilities (II) and personal strength (III) factors. Contrary to the present analysis, the authors did not find sufficient evidence for depicting the relating to others (I) factor as a core aspect of PTG. They highlight the importance of the ability to imagine a new way forward and, at the same time, their results suggest that greater personal strength might be more important for PTG than relationships with others. The least influential items appeared to be the items no. 1 (“My priorities about what is important in life”) and 2 (“An appreciation for the value of my life”), which partially corresponds with the findings from the presented analysis. According to Peters et al. (2021), who also examined the structure of the PTGI (Chinese adaptation, short version), the most central nodes were finding a new path in life, a greater sense of closeness with others, and the ability to do better things with life. In the network presented by those authors, the changing priorities item was very peripheral.

Based on the results of invariance testing, women had a higher average PTG score, which may be related to their higher emotionality and openness to communicate their own experiences (First et al., 2017). Another possible explanation is that women cope with the situation using more deliberative and reflective rumination, which might lead to higher posttraumatic growth (Vishnevsky et al., 2010). In general, emotion-focused coping strategies (positive reaction, acceptance, denial) are positively related to PTG (Butler et al., 2005; Helgeson et al., 2006; Prati and Pietrantoni, 2009).

Positive moderate correlations were observed between spirituality and posttraumatic growth. This result is in line with the theory of posttraumatic growth process as described by Tedeschi and Calhoun (1996) that spiritual change is one of the main aspects of PTG and is also supported by other studies (e.g., Prati and Pietrantoni, 2009).

Furthermore, weak positive correlations were observed between PTG and the scores of stressful events, as measured by the Life Stressor Checklist. As for individual types of stressors, weak to moderate correlations were found between PTGI score and the care for long-term sick loved ones, the (unexpected) death of loved ones and to be an accident witness or participant. Karanci et al. (2012) found that the type of event had a significant impact on only two domains of PTGI, namely the appreciation of life and the relating to others, e.g. an accident was more strongly correlated with the appreciation of life than with relating to others, and the unexpected death of a close person was strongly correlated with relating to others. Regarding trauma types, it seems that individuals who experienced interpersonal trauma (such as physical or sexual assaults) have more posttraumatic symptoms than those who experienced non-personal trauma (such as an accident or disaster), but no significant difference in PTG was found or there were only some variations in specific domains of PTG (Lowe et al., 2020; Thomas et al., 2021). We think that clinicians could benefit both from assessing posttraumatic stress symptoms (PTS) and signs of growth after trauma in their patients. Both PTS and PTG can be present and coexist; in such cases, PTG can be viewed more as an indicator of coping with PTS than as actual growth (Zoellner and Maercker, 2006; Thomas et al., 2021).

A weak negative correlation was observed between PTG and resilience. The evidence on this topic is inconsistent. While some authors found a positive relationship between these two variables (Bensimon, 2012), others detected a negative relationship (Levine et al., 2009). The inverse correlation between resilience and PTG could be explained by the fact that a more resilient person may not cognitively evaluate (cognitive processing plays a key role in PTG development; Tedeschi and Calhoun, 1996) a traumatic event as sufficiently threatening or disruptive and, as such, PTG may not develop (Levine et al., 2009). This is also in line with the suggestion of Westphal and Bonanno (2007) that resilient outcomes typically provide little need or opportunity for PTG. The results may also vary for a very pragmatic reason—the fact that different operationalizations of a construct could lead to different findings (see, e.g., Bensimon, 2012; Adamkovič et al., 2020).

Although we did not focus on the relationship between PTS and PTG in this study, this is a valuable topic, mainly from the therapeutic point of view. Studies have shown that there is a curvilinear relationship between PTS/PTSD and PTG, supporting the opinion that there cannot be PTG without some level of PTS (Sanki and O'Connor, 2021). The affective-cognitive processing model of PTG developed for mental health professionals takes the approach that PTS is a normal response to trauma and works with cognitions, appraisals, intrusions and emotional state and coping behaviors, until a reconciliation of pre- and post-assumptive worldview is completed (Joseph et al., 2012). The priority is to simply be present and non-judgmental and rather to support deliberative rumination to develop an individual pathway for PTG.

Psychotherapy constitutes a good context to explore positive changes in the aftermath of trauma. The simultaneous acknowledgement of patients’ suffering in a trustful and intimate therapeutic relationship enables them to explore positive changes as a result of their coping process. However, Zoellner and Maercker (2006) recall that the absence of growth should not be regarded as a failure, because PTG is not necessary for successful recovery from traumatic events.

## Limits and Perspectives of Further Research

The present study has several limitations. The first one regards the research sample. As the data comes from a representative sample of the adult Slovak population, the generalizability of the results to other cultural settings might be, obviously, limited. With regard to the representativeness of the sample, the participants are heterogeneous in terms of trauma profiles. Further research is needed to examine in detail the potential effect of the type of traumatic event on the structure of PTG. Second, the study was not focused on discussing the theoretical justification of the constitutive definition of PTG nor was it focused on qualitative analysis of items operationalization. Third, even though the study also presented a network analysis of PTG, it is important to acknowledge that this was not the original purpose of the study (the main aim of the study was to verify the psychometric properties and the factor structure of the PTGI) and, as such, the network presented herein has rather a demonstrative character. To learn more about the structure of PTG from a network perspective, more exclusive research on this topic would be needed. Ideally, such research would combine cross-sectional, longitudinal and experiential sampling design, while putting sufficient effort into having the study designs reasonably powered. The combination of between-person differences and within-person changes could help determine which aspects of PTG are more efficient to address by potential interventions or prevention programs. The present evidence, although based on cross-sectional data, suggests that intervening in one’s social relationships, self-confidence and communication training could promote PTG. Having a succinct PTG measure with a clear structure that produces a valid score across different cultures is, therefore, a necessity.

## Data Availability Statement

The datasets presented in this study can be found in online repositories. The names of the repository/repositories and accession number(s) can be found at: https://osf.io/kg5q8/.

## Ethics Statement

The studies involving human participants were reviewed and approved by Ethics Board of the Olomouc University Social Health Institute (OUSHI). The patients/participants provided their written informed consent to participate in this study.

## Author Contributions

BJ wrote the theoretical framework and discussion with inputs from NK and MA. MA performed statistical analyses. JH, PT, and NK supervised the study. All authors collectively conceived the main idea and the design of the study, have revised the manuscript, made a substantial contribution to this work, and approved it for publication.

## Funding

This work was funded by the Slovak Research and Development Agency (project no. APVV-17-0418 and APVV-20-0319).

## Conflict of Interest

The authors declare that the research was conducted in the absence of any commercial or financial relationships that could be construed as a potential conflict of interest.

## Publisher’s Note

All claims expressed in this article are solely those of the authors and do not necessarily represent those of their affiliated organizations, or those of the publisher, the editors and the reviewers. Any product that may be evaluated in this article, or claim that may be made by its manufacturer, is not guaranteed or endorsed by the publisher.

## Footnotes

^1^ https://osf.io/ucd7n/
